# Age at Onset Influences Progression of Motor and Non-Motor Symptoms during the Early Stage of Parkinson’s Disease: A Monocentric Retrospective Study

**DOI:** 10.3390/brainsci13020157

**Published:** 2023-01-17

**Authors:** Lanfranco De Carolis, Silvia Galli, Edoardo Bianchini, Domiziana Rinaldi, Manikandan Raju, Bianca Caliò, Marika Alborghetti, Francesco E. Pontieri

**Affiliations:** 1 Neurology Unit, NESMOS Department, Faculty of Medicine & Psychology, Sapienza—University of Rome, Sant’Andrea University Hospital, 00189 Rome, Italy; 2 Department of Clinical and Behavioral Neurology, IRCCS—Fondazione Santa Lucia, 00179 Rome, Italy

**Keywords:** age at onset, motor symptoms, non-motor symptoms, Late-Onset, Middle-Onset, Early-Onset, Parkinson’s disease

## Abstract

The interactions between the age at onset with other pathogenic mechanisms and the interplays between the disease progression and the aging processes in Parkinson’s disease (PD) remain undefined, particularly during the first years of illness. Here, we retrospectively investigated the clinical presentation and evolution of the motor and non-motor symptoms and treatment-related complications during the first 5 years of illness in subjects categorized according to age at onset. A total of 131 subjects were divided into “Early-Onset-PD” (EOPD; onset ≤49 years), “Middle-Onset-PD” (MOPD; onset 50–69 years) and “Late-Onset-PD” (LOPD; onset ≥70 years). The T0 visit was set at the time of the clinical diagnosis; the T1 visit was 5 years (±5 months) later. At T0, there were no significant differences in the motor features among the groups. At T1, the LOPD patients displayed a significantly higher frequency of gait disturbances and a higher frequency of postural instability. Moreover, at T1, the LOPD subjects reported a significantly higher frequency of non-motor symptoms; in particular, cardiovascular, cognitive and neuropsychiatric domains. The presented results showed a significantly different progression of motor and non-motor symptoms in the early course of PD according to the age at onset. These findings contribute to the definition of the role of age at onset on disease progression and may be useful for the pharmacological and non-pharmacological management of PD.

## 1. Introduction

Parkinson’s disease (PD) is a chronic progressive neurodegenerative disorder clinically diagnosed by the combination of bradykinesia with a resting tremor and/or rigidity in the absence of clinical or instrumental signs suggesting atypical or secondary parkinsonism [1]. Additional motor symptoms such as postural instability and gait disturbances may develop with the disease progression [1,2]. Most PD patients also experience a variety of non-motor symptoms [3], which may occur from the prodromal stage [4] and generally worsen in prevalence and severity along the disease course [5].

The symptomatic management of motor symptoms as well as a few non-motor symptoms of PD is based upon dopamine replacement therapy (DRT); in particular, levodopa [3]. After a few years of levodopa administration, however, patients develop complications with the features of motor and non-motor fluctuations and dyskinesia [3], which may further complicate the management and significantly aggravate independence and the quality of life [6]. Moreover, DRT may aggravate a few non-motor symptoms at a central as well as a peripheral level [7].

PD affects approximately 1% of people above 60 years of age; the disease prevalence increases with age. Indeed, age is a main risk factor for PD [3] and may affect the disease course in terms of the clinical, imaging, biological and therapeutic features [8,9,10,11]. However, interactions between the age at onset with other pathogenic mechanisms and the complex interplays between the disease progression and the aging processes remain to be defined, particularly during the first years of the disease course. The issue has been further complicated by a heterogeneity of the cut-off values for the categorization of the age of onset of PD; marked discrepancies are present in the literature [12,13]. According to a recent recommendation by the Early Onset Parkinson’s Disease Task Force of the Movement Disorder Society [14], PD patients may be categorized according to the age at onset into Early-Onset-PD (EOPD; onset ≤49 years of age), Middle-Onset-PD (MOPD; onset between 50 and 69 years of age) and Late-Onset-PD (LOPD; onset ≥70 years of age). Given these premises, in the present study, we retrospectively investigated the clinical presentation of PD and the evolution of the motor and non-motor symptoms and treatment-related complications during the first 5 years of the disease course in subjects categorized according to the age of onset as listed above [11,14]. We hypothesized that LOPD subjects would display a more frequent and rapid development of axial symptoms and a few non-motor symptoms with respect to the remaining groups. Based on this hypothesis, an older age at onset could be a relevant factor in the pharmacological and non-pharmacological management of PD patients in order to select more appropriate therapies, formulate a more accurate prognosis and manage the disabling symptoms and early complications of the disease more promptly and adequately.

## 2. Materials and Methods

This was an observational, retrospective and monocentric study, carried out on PD patients referred to the Movement Disorder Outpatient Service of the Sant’Andrea University Hospital, Sapienza University of Rome, in the period between 2013 and 2017. All subjects were diagnosed with PD according to the international criteria [1] and underwent scheduled follow-ups for at least 5 years. The protocol was waived by the local Ethical Committee, given the retrospective nature of the study.

The participants were divided into 3 groups according to the age at onset of the motor symptoms as follows:

- Early-Onset-PD (EOPD); onset ≤49 years;
- Middle-Onset-PD (MOPD); onset 50–69 years;
- Late-Onset-PD (LOPD); onset ≥70 years.

For each subject we selected the T0 (baseline) visit performed at the time of the diagnosis and T1 (follow-up), performed 5 years (±5 months) later.

The severity of the motor symptoms was evaluated by means of the Movement Disorder Society Unified Parkinson’s Disease Rating Scale Part 3 (MDS-UPDRS-III) [15]. In particular, the presence of tremors, postural instability and gait disorders was established by a score ≥1 from the sum of items 3.15, 3.16 and 3.17; ≥1 from item 3.12; and ≥1 from item 3.10, respectively. The non-motor symptoms were investigated by means of the Non-Motor Symptoms Scale (NMSS) [16] and considered to be present for a score ≥ 1 from each domain. The findings were reported in terms of the NMSS domain. Fluctuations were preliminarily screened by means of the Wearing-Off Questionnaire-19 (WOQ-19) [17,18]. Cases with WOQ-19 scores ≥2 were further evaluated in the search for a >20% difference in the MDS-UPDRS-III score between pre- and post-levodopa administration to confirm a fluctuation, as described previously [19]. The occurrence of dyskinesia was defined by a score ≥1 from items 4.1 and 4.2 of the MDS-UPDRS Part 4. In addition, we recorded the occurrence of falls within the observation period for each participant. For each subject, we calculated the total levodopa-equivalent daily dose (LEDD), levodopa-equivalent for dopamine agonist (DA LEDD) and monoamine oxidase-B inhibitors (iMAO-B LEDD) at T1.

The statistical analyses were performed using JASP 0.16.3 (JASP Team, University of Amsterdam), R v4.0.3 and RStudio v2022.02.3 + 492 for Windows (R Foundation for Statistical Computing, Vienna, Austria). The descriptive statistics were calculated for each variable. The continuous variables were expressed as the mean ± SD and the categorical variables as n (%). A chi-squared test was applied to assess the differences across the three age groups in terms of the demographic (gender) and clinical variables (presence/absence of tremors, postural instability, gait disturbances, motor fluctuations, dyskinesia and non-motor symptoms). Separate chi-squared tests were used as a post hoc analysis in the case of significant overall results. Yates’s continuity correction was applied if indicated. A Kruskal–Wallis test was used to compare the LEDD at T1 across the three groups and Dunn’s post hoc test was used in the case of significant results. McNemar’s test was used to assess the differences of frequency for tremors, postural instability and gait disturbances between T0 and T1 in the three groups. Bonferroni’s correction was applied to control the error for multiple comparisons. The significance threshold was set at α < 0.05.

## 3. Results

### 3.1. Demographic Features

**Figure 1:**
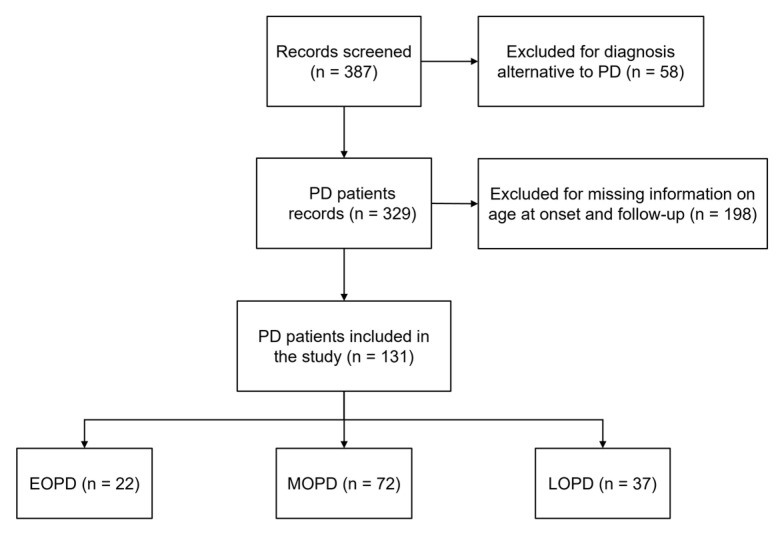
Flowchart of patient selection for the study. EOPD: Early-Onset-PD; MOPD: Middle-Onset-PD; LOPD: Late-Onset-PD; PD: Parkinson’s disease.

A total of 131 PD patients were included in the study based on the inclusion and exclusion criteria (Table 1). The flowchart of the patient selection is shown in Figure 1. There were 22 subjects (16.7%) in the EOPD group, 72 (54.9%) in the MOPD group and 37 (28%) in the LOPD group. The chi-squared test showed no significant difference in the sex distribution across the groups (χ^2^ = 3.057; *p* = 0.217).

### 3.2. Motor Symptoms

The chi-squared test showed no significant difference in the frequency of tremors, gait disturbances and postural instability across the three groups at T0 (Table 1). No patients reported FOG at T0. At T1, there was a significant difference across the groups for gait disturbances (*p* = 0.002) and postural instability (*p* = 0.006), with subjects in the LOPD group displaying a markedly higher prevalence than the other groups. In addition, a tendency toward a significant increase in an occurrence of falls within the first 5 years of the disease was found in patients with an older age at onset (*p* = 0.064). No significant differences were found for frequency of tremors, fluctuations, FOG and dyskinesia. No significant differences were found across the 3 groups with respect to the total LEDD at T1. The Kruskal–Wallis test showed a significant difference between the 3 groups for the iMAO-B LEDD and DA LEDD (*p* = 0.026 and *p* < 0.001, respectively), with the LOPD group displaying markedly lower values than the other groups. McNemar’s test showed a significant increase in the frequency of postural instability and gait disturbances between T0 and T1 in the MOPD (*p* = 0.003 and *p* = 0.001, respectively) and LOPD (*p* = 0.001 and *p* = 0.001, respectively) groups. Details of the results are shown in Table 1.

### 3.3. Non-Motor Symptoms

The chi-squared test showed significant differences across the three age groups for NMS1 (cardiovascular symptoms, including falls), NMS4 (perceptual problems and hallucinations) and NMS5 (attention and memory). Patients in the LOPD group reported symptoms more frequently for all these domains compared with the EOPD group and for NMS1 and NMS5 compared with the MOPD group. Moreover, a significant difference among the groups was found for NMS9 (the domain of miscellaneous symptoms, including pain, taste, smell, weight and sweating issues), with subjects in the MOPD and LOPD groups displaying a higher prevalence than the EOPD group (Table 2).

## 4. Discussion

In this retrospective, single-center and observational study, we investigated the progression of the motor and non-motor features and complications related to DRT during the first 5 years of illness in a cohort of subjects stratified according to their age at onset. The results demonstrated different patterns of progression of the motor and non-motor symptoms; subjects with LOPD displayed a more advanced occurrence of gait and postural disturbances together with cardiovascular, cognitive and perceptual alterations. Eventually, the NMS9 domain (pain, taste and smell, weight and sweating) was significantly more frequent in the LOPD and MOPD groups compared with the EOPD group (Table 1 and Table 2).

Regarding the motor symptoms, the prevalence of gait disturbances at T1 was significantly increased compared with T0 in both the MOPD and LOPD subjects (Table 1). An among-group analysis at T1 showed that the LOPD subjects displayed a significantly more frequent development of gait disturbances at T1 (65% compared with 32% and 27% in the MOPD and EOPD groups, respectively). Interestingly, the early occurrence of gait disorders in the LOPD group appeared to be primarily dependent on the disease process; the prevalence at the baseline (T0) was similar in the three groups. Similar findings were obtained for postural instability, although in this latter case the prevalence in the MOPD subjects was <20% (Table 1). These findings indicated a predictive role of a higher age at onset on the advanced development of gait disability as well as postural instability and a risk of falls in people with PD and should be considered when managing patients from the early disease stage, given the severe negative impact of gait and postural symptoms on daily functioning and disability. Moreover, they suggested an age-related stratification of subjects in clinical, pharmacological and rehabilitative trials and treatments. The present results were in line with previous reports [20] and confirmed that an older age at the onset of PD predicts an advanced impairment in balance and ambulatory functions. Conversely, we were unable to replicate the results of previous reports on advanced differences in the LEDD, FOG and dyskinesia prevalence between the EOPD, MOPD and LOPD groups during the first 5 years of illness [20,21,22]. These latter discrepancies might depend on the wide search for neurorehabilitation as well as different therapeutic strategies. Consistent with this hypothesis, we found significantly lower values for the DA LEDD and iMAO-B LEDD in the LOPD patients.

The LOPD patients also showed a significantly higher prevalence of several non-motor symptom domains (cardiovascular, including falls; perception/hallucinations; and memory/attention) compared with the other cohorts whereas a prevalence of the miscellaneous domain (NMS9) was similarly increased in the MOPD and LOPD groups after 5 years of illness. The features of the NMSS did not allow us to differentiate the direct effect of PD to those of comorbidities. Indeed, elderly subjects suffer more frequently from cardiovascular, osteoarticular (facilitating falls), cognitive and hallucinatory comorbidities and this possible bias should be considered. However, the more rapid and frequent development of these non-motor symptom domains in LOPD patients must be taken into account when evaluating the potential side-effects of different antiparkinsonian drug classes. Unfortunately, the NMSS was not routinely submitted at T0; thus, we could not exclude that the patients had already complained about cardiovascular/falls, cognitive or neuropsychiatric symptoms at the time of the PD diagnosis. However, the significantly higher occurrence of these non-motor symptom domains in the LOPD subjects encourages caution for the safety issues of treatment from the early disease stage.

This study had a few limitations. First, the retrospective nature, monocentric design and rather limited sample size might have hampered the generalizability of our results and limited the reliability of the evaluation during the 5-year period of the observation, facilitating a loss of specific information. However, we included subjects with a complete follow-up, evaluated regularly at 6–12-month intervals during all observation periods at the same movement disorder unit. Second, we collected the motor symptoms at the onset according to the reports of patients; this could have potentially reduced the accuracy of these data although the symptoms were confirmed and accurately objectivized at the first examination by an expert neurologist. Finally, we did not include general patient information such as education, occupation, income or marital status in our investigation. Similarly, we did not include data regarding adverse events (e.g., hospitalizations, drug-related adverse events, etc.). However, although these factors could influence the condition of patients, we designed our study to focus on the symptoms and levodopa-related complications (i.e., fluctuations and dyskinesia) without performing more advanced multivariate analyses. Considering the above-mentioned limitations, our data provided useful information on the progression profiles of symptoms as well as the differences between patients with a different age at onset during the first five years of the disease.

In conclusion, our findings indicated that the LOPD subjects were more prone to the development of motor and non-motor complications during the first 5 years of illness. Our presented results were in line with previous reports showing a faster progression of cognitive and neuropsychiatric (depression and anxiety) symptoms in subjects with LOPD compared with MOPD [11] and a lower burden of cognitive impairments in EOPD subjects compared with LOPD subjects [23]. Moreover, in accordance with our results, previous studies reported that patients with an older age at onset have a more rapid progression of motor and non-motor symptoms, more pronounced dopaminergic damage at DAT-Scan and a more pronounced α-synuclein and tau pathology [9,11]. Previously, it was hypothesized that there was a superposition of a topographic gradient of neurodegeneration related to the disease process and an aging-related temporal gradient of neuronal loss [8]. The above-described differences in the classically poor levodopa-responsive symptoms (such as gait and balance as well as cardiovascular, cognitive and neuropsychiatric features) between the LOPD subjects and patients with an earlier age at onset may support the previous hypothesis of a possible biological interaction of the aging process and disease-related neurodegeneration on the basal forebrain and brainstem non-dopaminergic structures [8]. Overall, our results support the concept that the age at onset might be relevant to stratify PD patients from an early stage of the illness. They also suggest that LOPD patients could be a more frail population that could need more careful management, including pharmacological therapies, rehabilitative treatments and caregiver assistance in order to avoid the advanced potential complications arising from the combination of disease and aging.

## Figures and Tables

**Table 1:** Demographics, motor features and LEDD of the investigated population and comparisons across age groups and between T0 and T1. Comparisons across the 3 groups are displayed on the horizontal axis (chi-squared and Kruskal–Wallis tests) and comparisons between T0 and T1 are displayed on the vertical axis (McNemar’s test). Significant results at baseline are highlighted in bold. Significant pairwise post hoc comparisons are shown by superscript letters, as indicated in the legend at the bottom of the table. SD: standard deviation; EOPD: Early-Onset-PD; MOPD: Middle-Onset-PD; LOPD: Late-Onset-PD; FOG: freezing of gait; LEDD: levodopa-equivalent daily dose; DA: dopamine agonist; iMAO-B: monoamine oxidase-B inhibitors; F: females; M: males; PD: Parkinson’s disease.

			EOPD (n = 22)	MOPD (n = 72)	LOPD (n = 37)	
**Age**			45.9 ± 3.5	61.3 ± 4.7	73.7 ± 3.6	
**Sex**	**F**		7 (32%)	26 (36%)	19 (51%)	χ^2^ = 3.057; *p* = 0.217
	**M**		15 (68%)	46 (64%)	18 (49%)	
**Tremor**		**T0**	16 (73%)	53 (74%)	26 (70%)	χ^2^ = 0.137; *p* = 0.934
		**T1**	18 (82%)	55 (76%)	26 (70%)	χ^2^ = 1.054; *p* = 0.590
			*p* = 0.500	*p* = 0.687	*p* = 1.000	
**Gait disturbances**		**T0**	2 (9%)	10 (14%)	10 (27%)	χ^2^ = 4.142; *p* = 0.126
		**T1**	**6 (27%) ^a^**	**23 (32%) ^a^**	**24 (65%)**	**χ^2^ = 12.904; *p* = 0.002**
			*p* = 0.219	*p* = 0.001	*p* = 0.001	
**Postural instability**		**T0**	0 (0%)	2 (3%)	3 (8%)	χ^2^ = 2.941; *p* = 0.230
		**T1**	**1 (5%) ^a^**	**13 (18%) ^a^**	**14 (38%)**	**χ^2^ = 10.147; *p* = 0.006**
			*p* = 1.000	*p* = 0.003	*p* = 0.001	
**Dyskinesias**		**T0**	-	-	-	-
		**T1**	4 (18%)	8 (11%)	7 (19%)	χ^2^ = 1.490; *p* = 0.475
**Fluctuations**		**T0**	-	-	-	-
		**T1**	9 (41%)	25 (35%)	9 (24%)	χ^2^ = 1.982; *p* = 0.371
**FOG**		**T0**	0	0	0	-
		**T1**	1 (5%)	5 (7%)	2 (5%)	χ^2^ = 0.213; *p* = 0.899
**Occurrence of falls**			1 (5%)	8 (11%)	9 (24%)	χ^2^ = 5.486; *p* = 0.064
**LEDD (mg)**			540 ± 308	525 ± 264	464 ± 134	χ^2^(2) = 1.641; *p* = 0.441
**DA LEDD (mg)**			**135 ± 117 ^a^**	**121 ± 102 ^a^**	**10 ± 43**	**χ^2^(2) = 35.600; *p* < 0.001**
**iMAO-B LEDD (mg)**			**73 ± 40 ^a^**	**61 ± 48 ^a^**	**38 ± 49**	**χ^2^(2) = 7.322; *p* = 0.026**

^a^ significant vs. LOPD.

**Table 2:** NMSS domains reported as present in the investigated population and comparisons across age groups at T1. Significant results of chi-squared test are highlighted in bold. Significant results at baseline are highlighted in bold. Significant pairwise post hoc comparisons are shown by superscript letters, as indicated in the legend at the bottom of the table. EOPD: Early-Onset-PD; MOPD: Middle-Onset-PD; LOPD: Late-Onset-PD; NMSS: Non-Motor Symptoms Scale; PD: Parkinson’s disease.

	EOPD (n = 22)	MOPD (n = 72)	LOPD (n = 37)	
**Cardiovascular, including falls (NMS1)**	**2 (9%) ^b^**	**9 (13%) ^b^**	**11 (30%)**	**χ^2^ = 6.315; *p* = 0.043**
**Sleep/fatigue (NMS2)**	13 (59%)	53 (74%)	25 (68%)	χ^2^ = 1.763; *p* = 0.414
**Mood/cognition (NMS3)**	13 (59%)	35 (49%)	14 (38%)	χ^2^ = 2.605; *p* = 0.272
**Perceptual problems/hallucinations (NMS4)**	**0 (0%) ^b^**	**5 (7%)**	**7 (19%)**	**χ^2^ = 6.878; *p* = 0.032**
**Attention/memory (NMS5)**	**1 (5%) ^b^**	**10 (14%) ^b^**	**12 (32%)**	**χ^2^ = 8.899; *p* = 0.012**
**Gastrointestinal tract (NMS6)**	3 (14%)	24 (33%)	14 (38%)	χ^2^ = 4.066; *p* = 0.131
**Urinary (NMS7)**	6 (27%)	20 (28 %)	12 (32%)	χ^2^ = 0.296; *p* = 0.863
**Sexual function (NMS8)**	3 (14%)	3 (4%)	1 (3%)	χ^2^ = 3.699; *p* = 0.157
**Miscellaneous (NMS9)**	**2 (9%) ^a,b^**	**28 (39%)**	**13 (35%)**	**χ^2^ = 6.911; *p* = 0.032**

^a^ Significant vs. MOPD; ^b^ significant vs. LOPD.

## Data Availability

The data presented in this study are available on request from the corresponding author.
